# Bartholin gland cyst: a clinical image

**DOI:** 10.11604/pamj.2022.43.174.38017

**Published:** 2022-12-05

**Authors:** Payal Bawankar, Ruchira Ankar

**Affiliations:** 1 Department of Medical Surgical Nursing, Smt Radhikabai Memorial College of Nursing, Datta Meghe Institute of Medical Sciences Deemed to be University, Sawangi (Meghe), Wardha, India

**Keywords:** Bartholin cyst, mucus-secreting gland, surgical intervention

## Image in medicine

Bartholin glands, also known as the greater vestibular glands, are a pair of 0.5 cm glands located in the lower right and left portions at the 4 o'clock and 8 o'clock positions of the vaginal introitus. The Bartholin gland is a mucus-secreting gland, which plays a role in vaginal lubrication. Bartholin glands are generally nonpalpable when not obstructed Bartholin cysts/abscesses are predominantly found in women of childbearing age. The incidence of Bartholin cysts is most often noted at the onset of puberty and increases with age until menopause. Symptomatic Bartholin cysts and abscesses account for 2 percent of all gynecologic visits per year. A 35-year-old patient was apparently all right 2 months back when she noticed a small cyst in the vaginal area which start increasing suddenly in 2-3 days due to obstruction of Bartholin gland which is not painful the patient admitted in a government hospital and after all investigation found that it is Bartholin cyst and surgical removal of Bartholin cyst done.

**Figure 1:**
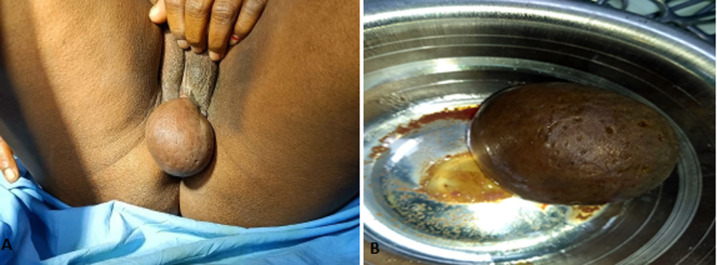
A) Bartholin gland cyst; B) surgical removal of Bartholin gland cyst

